# Nephrotoxicity of Phthalates: A Review Based on Epidemiological and Toxicological Evidence

**DOI:** 10.3390/toxics13110947

**Published:** 2025-11-03

**Authors:** Yuehang Wei, Minghui Zhang, Jiayuan Song, Tianyue Wang, Yuqin Ma, Liqiang Qin, Jiafu Li, Xiaoyan Qian, Jingsi Chen

**Affiliations:** 1 Department of Nutrition and Food Hygiene, Suzhou Medical College, Soochow University, Suzhou 215123, China; weiyuehang0205@163.com (Y.W.);; 2 Suzhou Industrial Park Centers for Disease Control and Prevention, Suzhou 215123, China

**Keywords:** phthalates, epidemiological study, nephrotoxicity, mechanism

## Abstract

Phthalates are a widely used class of plasticizers known to cause various health issues. Although numerous review articles have addressed the multi-organ toxicities of Phthalates and their metabolites, a specialized review focusing on their nephrotoxicity remains scarce. In this study, the nephrotoxicity of Phthalates and their metabolites is summarized from the views of epidemiological and toxicological evidence. Epidemiological studies have demonstrated a correlation between Phthalate exposure and abnormal urinary albumin-to-creatinine ratio (ACR) as well as glomerular filtration rate (eGFR) in children. In contrast, for adults, the epidemiological evidence for the association between Phthalates and ACR/eGFR remains controversial, necessitating further investigation. In this review, we explore the potential mechanisms by which Phthalates and their metabolites may induce nephrotoxicity. These mechanisms include the following: (1) induction of oxidative stress in renal cells; (2) reduction in aldosterone levels; (3) dysregulation of the renin-angiotensin system; (4) activation of endoplasmic reticulum (ER) stress; (5) renal fibrosis; (6) disruption of sodium and water homeostasis; and (7) activation of the heat shock response defense system. Finally, based on the current understanding, we propose future research directions and necessary efforts to advance knowledge in this field.

## 1. Introduction

Phthalates are a group of compounds derived from phthalic acid, primarily used as plasticizers to enhance the flexibility and softness of plastics [1]. They are widely applied in food packaging, chemical formulations, cosmetics, toys, furniture, flooring, wall coverings, and medical and electronic devices [2,3]. The global production of phthalates grew rapidly, increasing from 2.7 million tons in 2007 to 6 million tons annually by 2017 [4], representing more than 55% of the total global plasticizer consumption. The global consumption of phthalates was approximately 6 to 8 million tons per year [5].

Among numerous phthalates, seven homologs have been widely detected and studied and were commonly referred to as the “traditional phthalates”, including diethyl phthalate (2-ethylhexyl) (DEHP), butylbenzyl phthalate (BBP), dinoctyl phthalate (DNOP), dimethyl phthalate (DMP), diethyl phthalate (DEP), dibutyl phthalate (DBP), diisobutyl phthalate (DIBP), butyl benzyl phthalate (BBZP), and dioctyl phthalate (DOP). In recent years, several emerging phthalates have been identified, including diisononyl phthalate (DINP), diisodecyl phthalate (DIDP), and diphenyl phthalate (DPHP). These have been developed to replace traditional high-toxicity phthalates but have been less studied [6]. For example, six phthalates have been banned from children’s products, and China identifies DEP, DMP, and DNOP as environmental priority pollutants [7]. The EU restricted the use and SML of DBP, DEHP, BBP, DINP, and DIDP as early as 2007 [8], and similar measures were adopted by the United States, Israel, and Malaysia [9,10,11].

Phthalates are not chemically bound to the polymer system, which means that small changes in the environment can accelerate the leaching, migration, or vaporization from the plastic material into the surrounding environment. As a result, this has led to increased exposure and poses potential risks to human health [12]. Due to their mass production and widespread use in human society, phthalates have become one of the most serious environmental pollutants globally [13,14]. Therefore, the threats posed by phthalates to human health warrant greater attention.

Epidemiological and toxicological studies have demonstrated that phthalates exhibit a broad spectrum of toxic effects, including general toxicity, reproductive and developmental toxicity, hepatotoxicity, cytotoxicity, and nephrotoxicity [15,16]. The kidney is essential for fluid-electrolyte balance, acid-base regulation, waste excretion, and endocrine function via the renin-angiotensin-aldosterone system (RAAS) [17]. Given the central role of the kidney in systemic homeostasis, phthalate-induced nephrotoxicity represents a significant health concern that warrants further investigation.

This article summarized the nephrotoxic effects of phthalates and their metabolites in both humans and animals based on epidemiological and toxicological evidence. In addition, the potential mechanisms underlying phthalates-induced nephrotoxicity were reviewed, and insights and recommendations were offered for future research on this topic. This review mainly focuses on the nephrotoxic effects of traditional phthalates while also including evidence related to several emerging phthalate substitutes to provide a comprehensive understanding of both established and newly identified compounds. Although several reviews on phthalates exist [18,19,20,21], four issues remain: (1) evidence linking phthalate exposure to nephrotoxicity is inconsistent due to methodological and sample heterogeneity; (2) the pathogenic mechanisms remain unclear, and better exposure and evaluation models are needed; (3) oxidative stress is widely recognized but poorly characterized mechanistically, while other pathways are underexplored; and (4) toxicological data largely derive from animal models, with limited confirmation in humans.

## 2. Materials and Methods

There is currently a lack of systematic understanding and research on how phthalates can adversely affect the kidneys and even lead to kidney diseases. To further explore this yet-to-be-known field, firstly, we reviewed the current toxic effects of phthalates and analyzed the strengths and weaknesses of existing articles through the perspective of epidemiology. Secondly, we collected past literature on phthalates-induced nephrotoxicity from 1980 to 2025, narrowed the scope by searching for keywords such as “phthalates”, “nephrotoxicity”, “kidney injury”, “phthalates health risks”, “phthalates toxicity,” to comprehensively understand the current research status on the possible pathological mechanisms of phthalates-induced nephrotoxicity at home and abroad. We conducted a search across several databases, such as Web of Science, CNKI, PubMed, and ScienceDirect. This review is narrative in nature, aiming to summarize and integrate key epidemiological and toxicological findings rather than to conduct a formal systematic evidence synthesis. The drawings were created using Figdraw 2.0 (https://www.figdraw.com/, 13 September 2024).

### 2.1. Nephrotoxicity of PAEs

#### 2.1.1. Epidemiological Studies

In daily life, humans are exposed to phthalates through various sources such as food, air, and water, with these compounds being absorbed through the respiratory tract or intestine [22], eventually leading to renal abnormalities, including an increased albumin-to-creatinine ratio (ACR) and a decreased estimated glomerular filtration rate (eGFR) [23,24]. The epidemiological studies and available experimental evidence on the nephrotoxicity of phthalates are extensive and classified as follows.

#### 2.1.2. Adults

NHANES data (2009–2010) revealed associations between phthalate exposure and renal function in children and adolescents. Early findings in adults mainly came from occupational cohorts, whereas results in general populations were inconsistent, possibly due to renal compensatory capacity and heterogeneity in age, sex, and exposure levels. A Korean study further indicated that population characteristics modified these associations: females showed higher ACR and eGFR (5.4 mg/g and 105.5 mL/min/1.73 m^2^) than males (3.8 mg/g and 100.8 mL/min/1.73 m^2^), with ACR increasing and eGFR decreasing with age [25], findings that were consistent with those of Tsai et al. (2021) [26]. In contrast, Li et al. (2023) reported elevated ACR mainly in men after daily co-exposure to melamine and phthalates, suggesting combined pollutant effects and regional or ethnic variability [27]. A recent case–control study also found that phthalate exposure increased ACR and induced oxidative stress in patients with kidney stones [28].

Overall, epidemiological studies consistently linked phthalates and their metabolites to elevated ACR, while effects on eGFR remain uncertain. For example, Wang et al. (2022) found that phthalates and their metabolic byproducts were linked to higher eGFR in women but lower eGFR in men [29], which was consistent with the findings of Hong et al. [30]. Additionally, other studies indicated that MBZP and DEHP were positively correlated with ACR and negatively correlated with eGFR [31]. These conflicting results might be due to differences in the methods used for adjusting urinary creatinine levels. Many previous studies have adjusted urinary creatinine levels to control urinary dilution, and this traditional correction method may cause measurement errors [32,33], whereas Brien’s covariation-adjusted approach reduces interindividual variability and may provide a more accurate method for future studies [34].

#### 2.1.3. Fetal, Neonatal and Adolescent Periods

The pathophysiological mechanisms of Phthalate exposure had implications for pregnant women, who represented a particularly vulnerable population due to the susceptibility of the developing fetus [35]. Phthalates could cross the placental barrier during pregnancy, exposing the fetus and adversely affecting fetal and infant development, which could result in sustained intergenerational impairment [36,37,38]. According to Tsai et al., co-exposure to melamine and DEHP interacted significantly with NAG, a biomarker for early kidney injury, in pregnant women [26].

In 2011, phthalate contamination of food products in Taiwan became a major public health incident. DEHP and DINP were illegally used as emulsifiers, with concentrations reaching 2108 ppm and 8713 ppm in some probiotic supplements for children [39,40]. Although no direct toxicity was reported, previous animal studies had suggested that phthalates exposure could impact kidney function [41,42]. Since then, increasing evidence has linked phthalate exposure to altered ACR, eGFR, and microalbuminuria in children and adolescents. Microalbuminuria was identified as an important risk factor for cardiovascular disease and CKD [43,44]. Children with CKD are especially vulnerable due to repeated exposure from medical devices containing phthalates used for infusions and respiratory support [45].

Previous studies indicated that most Phthalate metabolites positively correlated with eGFR tended to be negatively correlated with ACR. Malits et al. (2018) showed that Phthalate metabolic byproducts such as MMP, MBP, MIBP, MECPP, MEHHP, MHXP, and MHPP were negatively linked to ACR in univariate analysis. In contrast, MMP, MEP, MIBP, MECPP, MEHHP, MEOHP, and MHPP were positively correlated with eGFR [32]. Liu et al. (2022) found a dose–response relationship between the increase in Phthalate metabolic byproducts, including MMP, MBP, MBZP, and MOP, and the decrease in eGFR in healthy children [23]. Jacobson et al. (2020) also identified a positive association between phthalate exposure and eGFR in children with CKD, but no link with ACR [46]. However, reverse causality likely influenced the positive correlations found between urinary chemicals and eGFR. Jin et al. (2018) suggested that reduced eGFR could have impaired the excretion of chemicals in urine, potentially leading to a misleading conclusion of a positive correlation between urine chemicals and eGFR [47]. Key epidemiological studies on phthalate exposure and renal biomarkers are summarized in Table 1.

### 2.2. In Vivo and In Vitro Experiments

There are various types of phthalates widely present in the external environment, posing not only adverse effects on human health but also significant harm to animals in the ecosystem. Currently, most animal models used in studies were mammals, such as rats and mice, although non-mammalian models, including quails and zebrafish, were also employed. The nephrotoxicity of phthalates was primarily evaluated using animal and in vitro models.

#### 2.2.1. *In Vivo* Experiment

Rats: The nephrotoxicity induced by phthalates has been well established, with early studies in rats demonstrating that exposure to DIBP and DBP resulted in abnormal body weight in the kidneys and other organs [53]. Exposure to BBP resulted in dilatation of the renal pelvis in rats fetuses [54]. David et al. reported that chronic exposure to DEHP aggravated chronic progressive kidney disease in male rats [55]. Subsequent studies showed that DEHP exposure led to damage in both body weight and kidneys in female rats, and also caused a reduction in nephron count, enlargement of glomerular volume, and a decrease in the size of Bowman’s capsules in their offspring, along with glomerular sclerosis, interstitial fibrosis, and loss of podocyte processes in adulthood [56]. DEHP also disrupted renal trace element balance [57].

Multiple studies demonstrated that DEHP induced oxidative stress in renal tissues, characterized by decreased peroxidase 1 (GPx1), superoxide dismutase (SOD), and glutathione (GSH) activities [58,59], accompanied by elevated Blood urea nitrogen (BUN) and creatinine levels [60]. Mixtures of phthalates caused renal tubular apoptosis and necrosis [61], and maternal DBP exposure led to renal fibrosis and downregulation of Fgf10/Fgfr2 in offspring [62,63]

Mice: An early study revealed that exposure to DEHP at concentrations ranging from 3000 to 12,000 ppm caused renal tubule damage in mice throughout 2 to 18 months [64]. Furthermore, DEHP exposure showed to induce chronic progressive kidney disease and tubular pigmentation in mice [55]. Subsequent experiments revealed that DEHP exposure led to glomerular atrophy, thickening of the glomerular basement membrane, renal tubule dilation, and enlargement of renal vesicles in mice [65]. Additionally, mice exposed to DEHP exhibited inflammation that contributed to kidney damage [63].

Numerous studies have demonstrated that DEHP exposure induced oxidative kidney damage in mice. Amara et al. found that DEHP exposure induced oxidative damage in mice by triggering an overproduction of reactive oxygen species (ROS), lipid peroxidation, protein carbonylation, and alterations in the activities of superoxide dismutase and catalase [66]. DEHP-induced oxidative stress resulted in excess oxygen species (ROS) production, lipid peroxidation, and reduced nuclear factor (Nrf2), oxygenase-1 (HO-1), and glutamate-cysteine ligase (GCLC) expression [67] These findings were consistent with earlier research by Erkekoglu [68]. Chronic exposure led to renal dysfunction and immune complex glomerulonephritis through peroxisome proliferator-activated receptor α (PPARα) activation [69]. and DEHP also triggered NLRP3 inflammasome activation and pyroptosis [70]. DBP exposure elevated serum creatinine and urea, while BBP disrupted renal structure and decreased BUN/CRE ratios [71].

Aquatic organisms: Aquatic organisms were exposed to phthalates through the aquatic environment. Research indicated that freshwater algae and cyanobacteria naturally produced monoethylhexyl phthalate (MEHP) or DBP and released these compounds into the aquatic environment [72]. In addition, phthalates in these environments were characterized by their low volatility, allowing them to persist and migrate across various aquatic species [73]. Phthalates were proven to be toxic to aquatic animals, and caused adverse effects on the immune system, metabolism, endocrine system, nervous system, genetic material, and development. These poisonous effects might lead to damage to organs and behavioral disorders [74].

Hu et al. (2016) conducted an analysis of 95 samples from wild marine aquatic organisms, such as fish, shrimp, and mollusks, collected from the East China Sea and the Yangtze River Delta [75]. The logKOW values of DBP and DEHP are 4.27 and 7, respectively [76]. In wild gilthead sea bream (S. aurata), BBP concentrations exceeded the LOQ, reaching 1.5 mg/kg and 0.33 mg/kg (wet weight) in samples from the Sousse and Monastir coasts, suggesting significant BBP contamination in seawater [71]. Additionally, enhanced renal phagocyte activity was observed in infected carp’s kidneys [77]. Exposure of halibut to DEP caused varying degrees of renal tissue and structural damage, such as tubular epithelial cell interpretation and multiple renal bleeding [78]. Moreover, Oya-Silva et al. (2023) reported that DIDP exposure in the neotropical catfish Rhamdia quelen increased DNA damage in head kidney cells and reduced antioxidant enzyme activity in tail kidney tissues [79].

Other animals: In addition to the commonly used animal models, studies also included species like quail. Ikele et al. (2011) observed the histopathological changes in the kidney caused by DEHP, including the destruction or fusion of Pyknotic nuclei, renal tubules, glomerular coagulation, and severe disruption of corpuscles [80]. Li et al. (2018) found glomerular contraction and tubular epithelial cell expansion in DEHP-exposed quail kidneys [81]. Wang et al. (2020) found that DEHP induced nephrotoxicity in quail by triggering the nucleoallogenic receptor (NXR) and regulating the cytochrome P450 system [82]. Representative toxicological studies on phthalate-induced nephrotoxicity are listed in Table 2.

#### 2.2.2. *In Vitro* Experiment

In vitro experiments, known for their specificity and simplicity, have emerged as a powerful adjunct technique for assessing chemical toxicity. MEHP exhibited significant nephrotoxic effects on cultured renal epithelial cells [88]. Ashari et al. (2020, 2022) confirmed that DEHP and MEHP triggered oxidative stress and cytotoxicity of Human Embryonic Kidney 293 Cells (HEK-293) cells [60,89]. DEHP exposure generated free radicals, leading to lipid peroxidation and altered activities of antioxidant enzymes such as SOD and catalase [66]. It also caused apoptosis in HEK-293 and human kidney epithelial (HK-2) cells, accompanied by glomerular hypertrophy, enhanced autophagy, and activation of inflammatory responses [63].

Several in vitro studies using rat renal tubular epithelial cells (NRK-52E) reported that DBP exposure triggered autophagy and promoted epithelial-mesenchymal transition (EMT) [85,90,91,92]. In contrast, Xie et al. (2023) conducted experiments with HK-2 cells and found that DBP exposure led to the induction of renal fibrosis [93]. The signaling pathways involved in DBP-induced fibrosis varied across studies and are discussed in detail in the nephrotoxicity mechanism section. DEHP exposure also induced renal fibrosis in NRK-52E cells [94,95]. In a different context, DBP exposure was found to induce oxidative stress in NRK 49F kidney cells, but this effect could be alleviated by the administration of vitamin C [96].

### 2.3. Possible Mechanisms of Phthalates Nephrotoxicity

The primary mechanisms through which phthalates induced nephrotoxicity encompassed various biological pathways. First, phthalates caused oxidative stress in renal cells, leading to damage to renal tubular cells and subsequent renal dysfunction. Second, phthalates were shown to reduce aldosterone levels, disrupting the balance of electrolytes and fluids in the body. Additionally, phthalates could aberrantly activate the renin-angiotensin system, which could elevate blood pressure and alter renal blood flow, thereby further impairing kidney function. Concurrently, endoplasmic reticulum stress and sodium-water retention in the kidneys were closely associated with the nephrotoxicity of phthalates, these factors exacerbated the burden on the kidneys, disrupted renal cell function, and ultimately resulted in kidney damage. Finally, although the activation of the heat shock response defense system was a protective mechanism, it might have been insufficient to effectively counteract the damage to renal cells induced by phthalates. In summary, phthalates impacted renal function through multiple mechanisms, resulted in the onset of nephrotoxicity. Consequently, this paper elaborated on the six distinct mechanisms outlined above.

### 2.4. Oxidative Stress

Many previous experiments confirmed that the nephrotoxicity of phthalates occurred through the induction of increased oxygen free radicals in the kidney or the reduction in antioxidant capacity and various antioxidant enzyme activities in the kidney [97,98,99,100]. This imbalance between oxidants and antioxidants led to a situation where the production of free radicals exceeded the scavenging capacity of SOD in the kidneys. Reactive oxygen species (ROS) generation was observed in both *in vivo* and *in vitro* studies. ROS promoted GSH synthesis by upregulating related enzymes, while elevated GSH inhibited the expression of the glutathione S-transferase (GST) gene [101]. These changes selectively regulated peroxidase gene transcription and further amplified ROS-driven oxidative stress.

**Figure 1:**
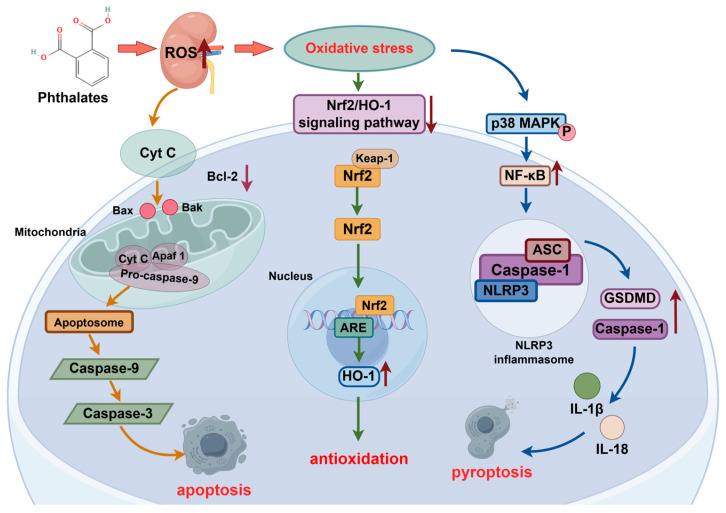
Potential Mechanisms of Phthalates-Induced Oxidative Stress in the Kidney. Abbreviations: Keap-1, Kelch-like ECH-associated protein 1; Nrf2, Nuclear factor erythroid 2-related factor 2; ARE, Antioxidant response elements; Cyt c, Cytochrome C; ROS, Reactive oxygen species; Bax/Bak, Bcl-2 protein family; Apaf1, Apoptotic protease activating factor 1; Caspase1/3/9, Caspase 1/3/9; NLRP3, Nod-like receptor family P3; ASC, Aptamer protein; GSDMD, Gasdermin D.

Drawing upon current research regarding the oxidative stress mechanisms associated with phthalates, we summarized three primary signaling pathways involved in oxidative stress, as illustrated in Figure 1: (1) Nrf2/HO-1 signaling pathway; (2) Bax/Caspase-3 signaling pathway; (3) Caspase/NLRP3 signaling pathway.

#### 2.4.1. Nrf2/HO-1 Signaling Pathway

The main cellular sensor of oxidative stress is the nuclear factor (Nrf2), located in the cytoplasm [102]. As a key regulator of redox reactions, Nrf2 plays an essential role in protecting against oxidative stress. Activators of Nrf2 significantly improve kidney function [103,104]. Nrf2 was crucial for the cellular defense mechanisms against oxidative stress, as it controlled the transcription of several protective factors through the antioxidant response element (ARE) [105,106]. Its activity is inhibited by Kelch-like ECH-associated protein 1 (Keap1), which promotes Nrf2 degradation under normal conditions [107,108]. Upon oxidative stress, Nrf2 stimulated HO-1, GSH, and catalase. The HO-1 enzyme catalyzed the conversion of heme into carbon monoxide, biliverdin, and iron ions, which were crucial in mediating oxidative stress and apoptosis [109,110].

Exposure to DEHP and DIBP elevates ROS levels and induces renal oxidative stress, accompanied by reduced Nrf2 and HO-1 expression, weakening the antioxidant Nrf2/HO-1 pathway [60,111]. Substances such as dimethyl fumarate and lycopene alleviate DEHP-induced oxidative stress by activating the Nrf2/HO-1 pathway and mitigating oxidative damage [112,113].

#### 2.4.2. Bax/Caspase-3 Signaling Pathway

The mitochondria is an essential organelle for maintaining cellular homeostasis and played a critical role in regulating apoptosis [114]. They are the primary source of ROS, and excessive ROS beyond antioxidant capacity leads to oxidative damage [115]. Mitochondrial dysfunction was a primary mechanism underlying chemical-induced nephrotoxicity [116,117]. Elevated ROS activates mitochondrial permeability transition pore (MPTP), causing swelling, membrane potential loss, and apoptotic factor release, which triggers apoptosis [118].

Apoptosis, a programmed form of cell death, is mainly mediated by the death receptor and mitochondrial pathways. These require activation of initiating caspases 8, caspases 9, caspases 10 and executioner caspase-3 [119]. The oxidative stress induced by phthalates led to apoptosis of renal cells via the mitochondrial pathway. This pathway was tightly regulated by members of the Bcl-2 protein family, which controlled mitochondrial membrane permeability and exhibited both pro-apoptotic and anti-apoptotic properties [120].

In rat kidneys, DEHP exposure disrupted redox balance, increased pro-inflammatory cytokines, NF-κB, and caspase-3, and decreased Bcl-2, confirming oxidative stress [121], aligning with the outcomes of other similar in vitro studies [89]. DEHP also activated the Bax/Caspase-3 pathway by promoting ROS, upregulating Bax and Caspase-3, and downregulating Bcl-2, leading to renal apoptosis [122]. In MEHP-exposed crucian carp kidney (CIK) cells, Cyt-c, Caspase 3, Caspase 9, and Bax expression increased, inducing apoptosis and autophagy [123].

Furthermore, DBP exposure was found to induce mitochondrial dysfunction, autophagy, apoptosis, and necroptotic cell death [124]. It elevated Bax, Caspase-8, cleaved Caspase-9, and Caspase-3, while reducing Bcl-2 and GLUT4 expression, promoting pancreatic β-cell apoptosis [125].

#### 2.4.3. NF-κB/Caspase/NLRP3 Signaling Pathway

Research indicated that oxidative stress and inflammatory responses were closely interconnected [126]. Pyroptosis, a type of programmed inflammatory cell death, was predominantly observed in endothelial cells and macrophages [127]. It includes classical and non-classical pathways. The classical pathway involves NLRP3 inflammasome activation of Caspase-1, which processes IL-1β and IL-18, initiating inflammation and cell damage through membrane rupture. In the non-classical pathway, lipopolysaccharide (LPS) directly activates GSDMD via Caspase-11 or Caspase-4/5, inducing apoptosis and inflammatory cascades [128,129,130].

Research showed that rats exposure to DEHP increased oxidative stress, activated NLRP3 inflammasome, and induced pyroptosis *in vivo* and *in vitro* [127]. Li et al. (2023) found that DEHP caused renal oxidative stress, activated Nrf2 and NLRP3 inflammasomes, triggered Caspase-1, and led to pyroptosis [113]. Co-exposure to polystyrene microplastics (PS-MPs) and DEHP aggravated renal injury in mice by reducing antioxidant enzymes, elevating heat shock proteins, and activating NF-κB/NLRP3 signaling, inducing apoptosis and inflammation [119]. Similarly, another study have demonstrated that DEHP exacerbated diabetes-induced kidney injury by mediating oxidative stress and activating the p38 MAPK/NF-κB pathway [131].

The p38 MAPK signaling pathway played a crucial role in regulating the downstream NF-κB pathway by promoting NF-κB nuclear translocation and influencing the expression of NLRP3 and associated inflammatory cytokines [132]. Exposure to DBP in mice and Caco-2 cells enhanced p38 MAPK phosphorylation and NF-κB expression, forming a positive feedback loop [133,134]. Co-exposure to DBP and BaP in rats also activated NLRP3 inflammasome, Caspase-1, and GSDMD, inducing apoptosis in renal tubular epithelial cells via TLR4/NF-κB signaling and increased IL-1β and IL-18 secretion [135]. Furthermore, DIBP exposure elevated IL-1β and TNF-α while suppressing IL-2, IFN-γ, Hepcidin 1, and β-defensin production. [111].

### 2.5. Reduction in Aldosterone Levels

Aldosterone is a hormone that promotes ion and water reabsorption in the kidneys, mainly acting on the distal nephron to enhance sodium retention and potassium excretion [136].Its synthesis is stimulated by angiotensin II (ATII) and potassium levels. The RAAS regulates ATII production, which activates angiotensin receptors AGTR1A and AGTR1B in the adrenal glomerular zone (ZG), initiating signaling pathways that promote aldosterone synthesis and adrenal cell proliferation [137,138]. CYP11B2, an enzyme essential for aldosterone synthesis, was specifically expressed in the adrenal glomerular zone (ZG), and its expression was enhanced by the activation of AGTR1 receptor signaling [139], and the reduction in CYP11B2 could lead to a decrease in aldosterone levels.

Previous studies confirmed that aldosterone could induce the activation of mineralocorticoid receptor (MR) in stromal cells [140]. Additionally, studies showed that intrauterine exposure to DEHP could decrease renal serum aldosterone levels and MR expression in stromal cells [141]. Martinez-Arguelles et al. (2014) further found that in-utero DEHP exposure in utero impaired the regulation of endogenous aldosterone, diminishing potassium channel regulation and decreasing the expression of ATIIRs. DEHP might have targeted the peroxisome PPAR pathway, influencing the expression of PPARα and PPARδ [142]. The PPAR pathway is a sensitive target of phthalates, and other PPAR family members also influence adrenal steroidogenesis. In particular, PPARγ regulates aldosterone biosynthesis by modulating CYP11B2 expression in H295R [143] and HAC15 cell lines [144].

### 2.6. Abnormal Activation of Renin-Angiotensin System

The renin-angiotensin system (RAS) played an essential role in regulating water and electrolyte balance, plasma concentration, and extracellular fluid volume [145]. Angiotensinogen, synthesized by the liver, was cleaved by the renin—produced by proximal convoluted tubule cells—into angiotensinI (AngI). AngI was subsequently converted into its active form, angiotensinII (AngII), by the action of angiotensin-converting enzyme (ACE). AngII was a multifunctional effector molecule that induced smooth muscle contraction and exerts vasoactive effects on all blood vessels [146]. Elevated AngII levels cause renal vasoconstriction and reduce the glomerular filtration area, leading to proteinuria. It also promotes renal hypertrophy, inflammation, and fibrosis via transforming growth factors, ultimately resulting in glomerular fibrosis and sclerosis. During nephrogenesis, AngII regulates ureteric bud (UB) branching through AT1R and AT2R, directly stimulating Pax2 and GDNF expression in UB branches to influence nephron formation [147,148]. Simultaneously, PPARα ligands had been shown to inhibit RAS activity by decreasing the expression of AT1R [149,150], and suppressing AngII-mediated signaling pathways. This included the phosphatidylinositol 3-kinase and mitogen-activated protein kinase pathways [151], as well as toll-like receptor 4-dependent signaling pathways [152], these actions likely contributed to the inhibition of RAS [153].

**Figure 2:**
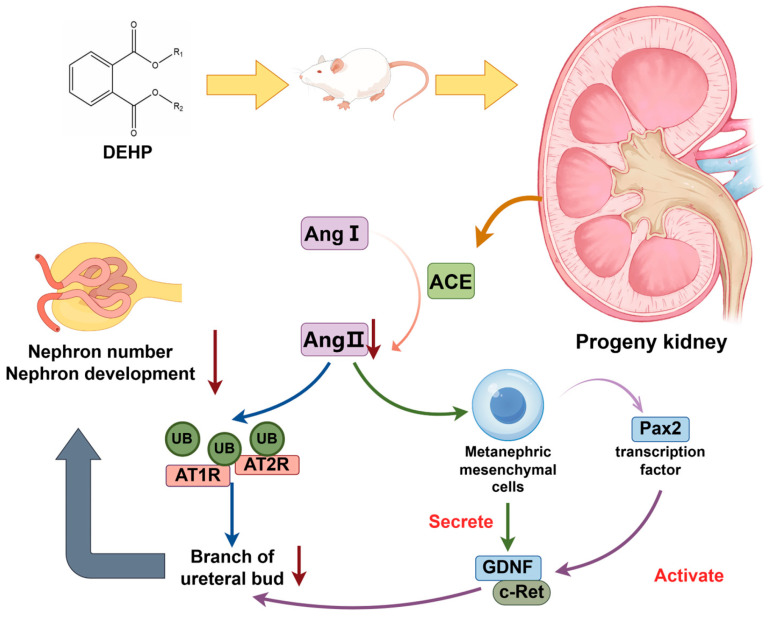
Mechanisms Underlying DEHP-Induced Abnormal Activation of Renal RAS. Abbreviations: ACE, Angiotensin enzyme; Ang I/Ⅱ, AngiotensinⅠ/Ⅱ; Pax2, Transcription factor; GDNF, Glial cell line-derived nerve growth factor; C-ret, C-retrovirus; UB, Ureteric bud; AT1R/AT2R, Angiotensin II type 1/type 2.

Lee et al. (2016) found that maternal DEHP exposure enhanced the expression of AT1R in offspring [154]. Conversely, DEHP reduced aldosterone levels by downregulating the ACE-AngII-AT1R pathway, which regulates sodium reabsorption and maintains sodium-potassium homeostasis. This pathway also contributes to renal inflammation and fibrosis [155,156,157]. Furthermore, other study confirmed that maternal exposure to DEHP reduced renin and AngII expression levels in offspring [158]. The mechanistic pathways and effects are illustrated in Figure 2.

### 2.7. ER Stress

In biological cells, the endoplasmic reticulum (ER) was essential for the synthesis, folding, and assembly of intracellular transmembrane proteins, steroids, and lipids. Most proteins were synthesized and folded within the endoplasmic reticulum [159,160]. Recent studies indicated that renal disorders, such as diabetic nephropathy, kidney fibrosis, and ischemia-reperfusion injury, were primarily associated with ER stress [161]. Furthermore, ER stress has been shown to exacerbate glomerular and tubular injury in these conditions [162].

**Figure 3:**
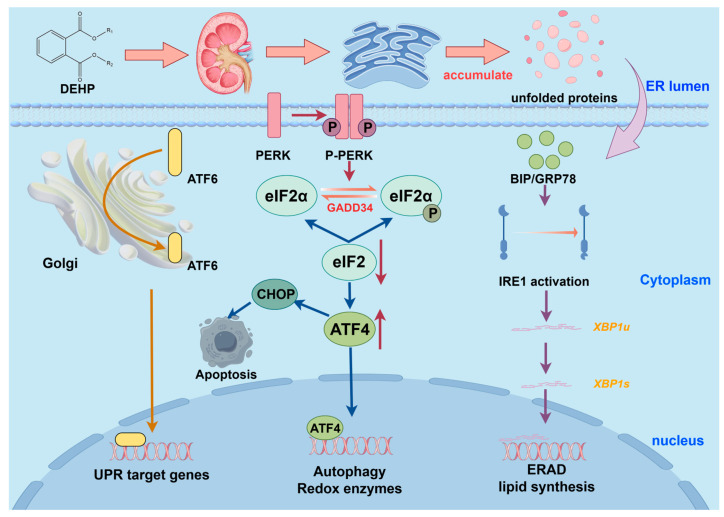
Potential Mechanisms of DEHP-Induced Endoplasmic Reticulum Stress in the Kidney. Abbreviations: BIP/GRP78, Binding immunoglobulin protein/Glucose-regulated protein 78; IRE1, Inositol requiring enzyme 1; XBP1, X box binding protein 1; ERAD, Endoplasmic reticulum associated degradation; PERK, Endoplasmic reticulum kinase PERK; eIF2α, Eukaryotic translation initiation factor 2α; GADD34, Apoptosis inducing factor 34; ATF4/6, Activating transcription factor 4/6; CHOP, Apoptosis promoting factor.

Recent scientific research revealed that DEHP could trigger the unfolded protein response (UPR) and ER stress, potentially adversely affecting protein metabolism in various organisms [163].During ER stress, unfolded proteins cause dissociation of chaperones such as binding immunoglobulin protein (BIP) and glucose-regulating protein GRP78, leading to phosphorylation and dimerization of IRE1 and PERK, while ATF6 translocates from the ER membrane to the Golgi apparatus. These processes activate the ER-associated degradation (ERAD) pathway and reduce protein influx by suppressing translation efficiency [164,165]. Following DEHP exposure, cells exhibited a pronounced ER stress response, marked by a notable rise in the levels of GRP78 and GRP94 [166]. In this biological context, X-box binding protein 1 (XBP-1) played a crucial regulatory role, especially in orchestrating the UPR mechanisms [167]. Under stress conditions, unspliced XBP-1 (XBP-1u) is converted into its active spliced form (XBP-1s), which upregulates ER chaperone expression [168]. This process is summarized in Figure 3.

### 2.8. Renal Fibrosis

Several studies have shown that DBP exposure can induce epithelial–mesenchymal transition (EMT) in experimental animals. Transforming growth factor-beta (TGF-β) is a key cytokine driving EMT by disrupting epithelial polarity and adhesion, which contributes to renal fibrosis [91,96]. Notably, the mechanisms underlying renal fibrosis induction appear to differ across studies.

DBP exposure was found to enhance the expression of Connexin 43 (Cx43) by activating the AngII/AMPKα2 signaling pathway. AMPKα2, a crucial regulatory enzyme within the AMP-activated protein kinase (AMPK) family, plays an important role in EMT and has been recognized as a key regulator in this process [169]. Cx43 is a protein that facilitates intercellular communication through gap junctions, and previous studies have suggested Cx43 as a promising target for renal fibrosis therapy [170]. Xie et al. (2023) have demonstrated that DBP promotes renal fibrosis by activating the AngII/AMPKα2/Cx43 pathway and inducing EMT in renal tubular epithelial cells (RETC), thus highlighting DBP as a potential therapeutic target for renal fibrosis [93].

Ye et al. (2020) reported that maternal DBP exposure induced EMT in offspring renal tubular epithelial cells via the RhoA/ROCK pathway, leading to fibrosis. In HR-52E cells, DBP exposure decreased E-cadherin and increased α-SMA expression, confirming EMT induction [90].

Zhao et al. (2020) found that DBP also affected the Hedgehog signaling pathway, which regulates development and regeneration. DBP exposure caused overexpression of HhIP, a pathway inhibitor, thereby suppressing Hedgehog activity, impairing kidney development, and promoting autophagy that may contribute to fibrosis [92].

Exposure to DEHP (10–25 μM) induced morphological changes and EMT in renal proximal tubular cells, accompanied by PPAR downregulation. These findings suggest that DEHP exposure may aggravate renal fibrosis and nephropathy, particularly in individuals with existing kidney disease [95]. Moreover, in a study conducted by Zhang et al. (2022), exposure to MEHP has been shown to cause oxidative damage in hepatocytes, which subsequently led to the upregulation of key molecules involved in fibrosis. This process ultimately resulted in the development of liver fibrosis in rats [171].

### 2.9. Sodium and Water Retention

Sodium and water retention fundamentally arose from an imbalance in internal and external fluid exchange, primarily due to a disorder in renal regulatory function [172]. This condition arises from reduced eGFR or increased tubular reabsorption of sodium and water, causing extracellular sodium accumulation [173]. Sodium transporters were known to play a crucial role in sodium-water imbalance [174], mainly including sodium-potassium-chloride cotransporter 2 (NKCC-2), sodium/proton exchanger 3 (NHE-3), and sodium-potassium ATPase (Na+/K+-ATPase). Adolescent exposure to phthalates has been shown to upregulate NHE-3 protein levels in the distal renal tubules of mouse offspring [175]. Studies reported that DEHP exposure markedly reduced urinary sodium excretion in rats, possibly due to altered aldosterone metabolism or Na+/K+-ATPase inhibition [57]. It was noteworthy that the kidneys of very preterm infants possessed immature mechanisms for sodium and water retention, potentially leading to delayed toxic effects after DEHP exposure [176].

### 2.10. Activate Heat Shock Response Defense System

The heat shock response (HSR) is activated by increased expression of heat shock proteins (HSPs) following exposure to environmental stressors. HSPs, highly conserved stress-related molecules, mediate inflammatory processes via the NF-κB signaling pathway and are markedly upregulated in damaged tissues [177,178]. Li et al. (2018) found that DEHP exposure in quails caused kidney injury associated with altered HSF-regulated HSP expression, indicating activation of the HSR defense mechanism [81]. Recent studies have demonstrated that DEHP exposure activated the NF-κB /NLRP3 signaling pathway, stimulated heat shock protein levels, and induced pyrodeath [119].

## 3. Conclusions and Outlook

Phthalates and their metabolites are widespread in various environmental media and pose potential risks to renal health. Current evidence suggests that their nephrotoxicity is multifactorial, mainly involving oxidative stress, dysregulation of the renin–angiotensin system, and fibrosis processes, which collectively impair renal structure and function. However, inconsistencies remain between epidemiological and toxicological findings due to variations in exposure levels, metabolite types, and study populations.

(1) Although oxidative stress and endoplasmic reticulum stress have been consistently identified as key mechanisms, further research is required to elucidate other molecular pathways and the relevance of animal findings to humans. The role of novel phthalates and their metabolites also warrants attention, as restrictions on traditional phthalates have led to the emergence of substitutes whose toxicological profiles are not yet fully understood.

(2) Future research should focus on population heterogeneity, including genetic susceptibility, age, sex, and lifestyle factors, which may influence phthalate metabolism and renal vulnerability. Building multifactorial epidemiological models could improve the assessment of nephrotoxic risks across diverse populations.

(3) Long-term ecological and health risk assessments of both traditional and alternative plasticizers are essential. Establishing sustainable environmental monitoring and evaluating bioaccumulation and transformation pathways will provide a scientific basis for public health protection. In addition to strengthening regulatory controls, efforts should also emphasize the recycling and safe disposal of existing phthalate-containing products to reduce the demand for new production and minimize environmental accumulation. Given the growing evidence of renal toxicity, global regulatory efforts should be strengthened to control the use of phthalates in food-contact materials and medical devices.

## Figures and Tables

**Table 1:** Epidemiological evidence linking urinary phthalate exposure to renal markers.

Phthalate Exposure	ACR	eGFR	References
MECPP, MEHHP, MBP, MBzP	→	↑ (women) ↓ (men)	Hong et al., 2024 [30]
Melamine + MBP, MEP, MBzP, MECPP, MEHHP	↑ (men) → (women)	–	Li et al., 2023 [27]
MBzP, MCPP, MEOHP, MEP, MEHP, MNP, MiBP	–	↑ (women) ↓ (men)	Wang et al., 2022 [29]
MBzP, MCPP(HMW)	–	↓	Liu et al., 2022 [23]
MMP, MBP, MBzP, MOP	–	↓	Liu et al., 2022 [48]
Melamine + MMP, MEHHP, MEOHP, MECPP/BBzP, MBzP	↑	↑	Tsai et al., 2021 [26]
MBP, MiBP, MBzP, MECPP, MEHHP	↑	↓	Kang et al.,2021 [31]
DEHP, DBP, BBP	↑	↓ (men)	Lee et al., 2020 [25]
MECPP, MEHHP, MBzP	↑	–	Chen et al., 2020 [49]
∑LMW phthalates	→	↑	Jacobson et al., 2020 [46]
MBP, MiBP, MBzP, DEHP	↑	–	Kang et al., 2019 [50]
MMP, MBP, MiBP, MECPP, MEHHP	↓	↑	Malits et al., 2018 [32]
Modelled phthalate exposure	→	→	Jin et al., 2018 [47]
DEHP	↑	–	Tsai et al., 2016 [51]
MEHHP, MEHP, MECPP, DIDP(HMW)	↑	–	Trasande et al., 2014 [52]

Direction symbols: ↑ = positive association (marker increases with phthalate load), ↓ = negative association, → = null/inconsistent, – = not assessed.

**Table 2:** Classification of phthalate-induced nephrotoxicity by compound and reference.

Phthalate(s)	Species	Observed Renal Effects	Mechanism or Markers Involved	References
DEHP	Rat	Kidney injury, fibrosis, loss of podocyte processes	Oxidative stress, ↑ BUN/CRE, ↓ GSH, SOD, GPx1	David et al., 2000; Wei et al., 2012; Erkekoglu et al., 2010, 2014; Ashari et al., 2020 [55,56,58,59,60]
	Mouse	Tubular damage, CKD, inflammation	↓ Nrf2, HO-1, GCLC, GSH	Ward et al., 1986; Jiang et al., 2021; Amara et al., 2019, 2020 [64,65,66,67]
	Mouse	Immune complex glomerulonephritis	PPARα pathway, NLRP3 activation	Kamijo et al., 2007; Tang et al., 2019 [69,70]
	Aquatic organism	Kidney hemorrhage, epithelial damage	Lipid peroxidation, ROS, ↓ antioxidants	Xiao et al., 2018 [78]
	Quail	Glomerular contraction, tubular expansion	PXR activation, cytochrome P450	Ikele et al., 2011; P. Li et al., 2018; Wang et al., 2020 [80,81,82]
DIBP, DBP	Rat	Renal fibrosis; reduced Fgf10 and Fgfr2	↓ Fgf10/Fgfr2 signaling pathway	White et al., 2009; Jiang et al., 2015; Sun et al., 2018 [83,84,85]
	Mouse	Elevated creatinine and urea; kidney tissue damage	↑ Bax, ↓ Bcl-2, ↓ Bax/Bcl-2	Liang et al., 2021 [86]
BBP	Rat	Fetal renal pelvis dilatation	–	Ema et al., 1992 [54]
		Distal tubule and collecting duct damage	↑ urinary excretion of 2OHE1 ↓ urinary excretion of E4	Nakagomi et al., 2018 [61]
	Mouse	Kidney tissue changes	↑ AST, LDH	Beltifa et al., 2017 [71]
	Aquatic organism	BBP accumulation in tissues	short-branched MPEs, ↑ AST, LDH	Hu et al., 2016; Beltifa et al., 2017 [71,75]
DIDP	Aquatic organism	DNA damage in head kidney cells	↓ antioxidant enzymes	Oya-Silva et al., 2023 [79]
DEP	Aquatic organism	Tubular epithelial cell interpretation; hemorrhage	–	Xiao et al., 2018 [78]
Mixed	Rat	Apoptosis and necrosis in renal tubular epithelial cells	–	Dai et al., 2020 [87]

Direction symbols: ↑ = positive association (marker increases with phthalate load), ↓ = negative association, – = not assessed.

## Data Availability

No new data were created or analyzed in this study. Data sharing is not applicable to this article.

## Abbreviations

Abbreviation	Full Term
ACE	Angiotensin-converting enzyme
ACR	Albumin/creatinine ratio
AMPK	Adenosine 5‘-monophosphate-activated protein kinase
ASC	Aptamer protein
ATF4/6	Activating transcription factor 4/6
Bax	Bcl-2 protein family
BBP	Butylbenzyl phthalate
BBZP	Butyl benzyl phthalate
BIP	Binding protein
BUN	Blood urea nitrogen
CHOP	C/EBP homologous protein
CKD	Chronic kidney disease
DBP	Dibutyl phthalate
DEHP	diethyl phthalate (2-ethylhexyl)
DEP	Diethyl phthalate
DIBP	Diisobutyl phthalate
DIDP	Diisodecyl phthalate
DINP	Diisononyl phthalate
DMP	Dimethyl phthalate
DNOP	Dinoctyl phthalate
DOP	Dioctyl phthalate
DPHP	Diphenyl phthalate
eGFR	Estimated Glomerular Filtration Rate
EMT	Epithelial-mesenchymal transition
ER	Endoplasmic reticulum
ERAD	Endoplasmic reticulum associated degradation
GCLC	Glutamate-cysteine ligase
GDNF	Glial cell line-derived nerve growth factor
GLUT 4	Glucose transporter 4
GPx1	Glutathione Peroxidase 1
GSH	Glutathione
HO-1	Heme oxygenase-1
HSR	Heat shock response
Keap1	Kelch-like ECH-associated protein 1
LPS	Lipopolysaccharide
MR	Mineralocorticoid receptor
NF-κB	Nuclear factor kappa-light-chain-enhancer of activated B cells
NLRP3	Nod-like receptor family P3
Nrf2	Nuclear factor erythroid 2-related factor 2
NXR	Nucleoallogenic receptor
OPEs	Organic phosphate esters
PFASs	Polyfluorinated substances
PPARα	Peroxisome proliferator-activated receptor α
PS-MPs	Polystyrene microplastics
RAAS	renin-angiotensin-aldosterone system
RAS	Renin-angiotensin system
ROS	Reactive oxygen species
SOD	Superoxide Dismutase
UB	Ureteral bud
UPR	Unfolded protein response
XBP1	X box binding protein 1
ZG	Glomerular zone
